# Evaluating Genetic Regulators of MicroRNAs Using Machine Learning Models

**DOI:** 10.3390/ijms26125757

**Published:** 2025-06-16

**Authors:** Mert Cihan, Uchenna Alex Anyaegbunam, Steffen Albrecht, Miguel A. Andrade-Navarro, Maximilian Sprang

**Affiliations:** 1 Institute of Organismic and Molecular Evolution, Faculty of Biology, Johannes Gutenberg University Mainz, 55128 Mainz, Germany; mercihan@uni-mainz.de (M.C.); andrade@uni-mainz.de (M.A.A.-N.); 2 Department of General Practice and Primary Health Care, Faculty of Medical and Health Sciences (FMHS), The University of Auckland, Auckland 1023, New Zealand; 3 Institute of Quantitative and Computational Biology, Johannes Gutenberg University Mainz, 55128 Mainz, Germany; 4 Department of Dermatology, University Medical Center of the Johannes Gutenberg-University Mainz, 55131 Mainz, Germany

**Keywords:** microRNA, machine learning, gene expression modeling, regulatory networks, functional genomics

## Abstract

This study explores the genetic regulators of microRNAs (miRNAs) using a set of machine learning models to predict miRNA expression levels from gene expression data. Employing machine learning, we accurately predicted the expression of 353 human miRNAs (R^2^ > 0.5), revealing robust miRNA–gene regulatory relationships. By analyzing the coefficients of these predictive models, we identified genetic regulators for each miRNA and highlighted the multifactorial nature of miRNA regulation. Further network analysis uncovered that miRNAs with higher predictive accuracy are more densely connected to their top predictive genes, reflecting strong regulatory control within miRNA–gene networks. To refine these insights, we filtered the miRNA–gene interaction networks to identify miRNAs specifically associated with enriched pathways, such as synaptic function and cardiovascular processes. From this pathway-centric analysis, we present a curated list of miRNAs and their genetic regulators, pinpointing their activity within distinct biological contexts. Additionally, our study provides a comprehensive set of metrics and coefficients for the genes most predictive of miRNA expression, along with a filtered subnetwork of miRNAs linked to specific pathways and phenotypes. By integrating miRNA expression predictors with network analysis and pathway enrichment, this work advances our understanding of miRNA regulatory mechanisms and their roles across distinct biological systems. Our approach enables researchers to train custom models using TCGA data and predict miRNA expression from gene expression inputs.

## 1. Introduction

MicroRNAs (miRNAs) play a critical role in the regulation of gene expression by binding to target messenger RNAs (mRNAs) and either promoting their degradation or inhibiting their translation [1]. These small, non-coding RNAs are involved in a wide array of cellular processes, including development, differentiation, and apoptosis, making them essential for maintaining cellular homeostasis [2,3].

Accurate profiling of miRNA expression is crucial for understanding miRNA functions. To predict miRNA targets, among other methods, the negative correlation between miRNA and mRNA expression is used to identify potential novel miRNA target binding sites on genes [4,5,6]. By mapping these interactions, researchers can elucidate how miRNAs influence various cellular pathways and processes, highlighting their potential as therapeutic targets and biomarkers [7,8]. This characterization not only relies on direct binding site identification but also on integration with annotation databases, high-throughput experimental validation, evolutionary conservation studies, and network-based analysis [3,9,10,11,12].

Quantifying miRNA expression remains challenging due to biases in current experimental methods. Small RNA-seq often relies on adapter ligation and PCR amplification, introducing representation biases that affect accuracy and reliability [13,14,15]. Issues like false high fold changes from low expression values and alignment errors also arise, especially when compared to the higher precision of qRT-PCR [16]. Moreover, integrating data across platforms is complicated by differing biases and error profiles. A major limitation is the lack of validated reference controls for normalization, leading to variability and poor cross-study comparability [13,17,18,19].

To address the limitations in miRNA expression quantification, a range of machine learning and computational methods have been developed. For instance, a constrained least squares approach has been reported for imputing missing miRNA expression values, improving data completeness in partially observed miRNA matrices [20]. Moreover, MMpred employs regression to predict miRNA expression from microarray data, facilitating the inference of miRNA–mRNA interactions [4]. miREACT utilizes motif enrichment analysis to estimate miRNA activity from single-cell RNA-seq data, providing insights into miRNA regulation at the single-cell level [21]. Similarly, miRSCAPE leverages tree-based machine learning to infer miRNA expression from single-cell RNA-seq data, enabling the study of miRNA activity in contexts where direct measurement is challenging [22]. Other frameworks aim to infer miRNA activity or regulatory influence, such as the enrichment-based method for estimating miRNA repression from gene expression profiles [23] and the causal inference approach for detecting miRNA–mRNA regulatory relationships directly from expression data [24]. Collectively, these methods underscore the versatility of machine learning in tackling both expression-level and functional characterization challenges in miRNA biology.

While mRNA data is used to infer miRNA expression, the genetic regulators of miRNAs remain poorly understood as even intronic miRNAs often show weak correlation with their host genes [25,26]. Moreover, no existing tool offers pretrained models that allow direct inference of miRNA expression from bulk RNA-seq input alone, limiting broader applicability.

In this study, we use gene expression data from RNA sequencing to predict miRNA expression levels, offering an approach that leverages the correlations between gene and miRNA expressions to build predictive machine learning models, providing a more accessible and accurate computational alternative to direct miRNA measurement. By doing so, it allows us to infer miRNA activity and its regulatory impact on genes, facilitating deeper insights into both cellular mechanisms and disease pathways. To this end, we applied ridge regression [27], a regularization technique suited for handling multicollinearity and high-dimensional data, to predict miRNA levels from RNA-seq data obtained from The Cancer Genome Atlas (TCGA) [28] from both normal and cancer tissues across thousands of samples. By analyzing the regression coefficients, we identified predictive genes for each miRNA considered, revealing key regulatory elements within the gene–miRNA network. Subsequent network analysis, incorporating miRNA binding data, enabled us to map out intricate interactions and pathways to characterize the functional relevance and biological implications of these predictive genes. Our approach is the first to provide pretrained, reproducible models that directly infer miRNA expression from bulk RNA-seq data while simultaneously uncovering gene-level regulators—offering a framework to explore miRNA control across diverse tissues and disease contexts.

## 2. Results

In this study, we applied ridge regression to develop a set of models to predict miRNA expression levels from RNA sequencing data. By leveraging the correlations between gene and miRNA expressions, our approach provides a computational alternative to direct miRNA measurement. Additionally, we constructed a network of miRNAs and their target genes, integrating experimentally validated interactions and predicted conserved interactions to understand the functional relevance and biological implications of these regulatory relationships better. We then used the feature coefficients from these models to identify key predictive genes, allowing us to explore the regulatory elements within the gene–miRNA network. This analysis offers a deeper insight into miRNA–gene interactions and their roles in cellular mechanisms and disease pathways.

### 2.1. Model Development and Performance Assessment

**Figure 1:**
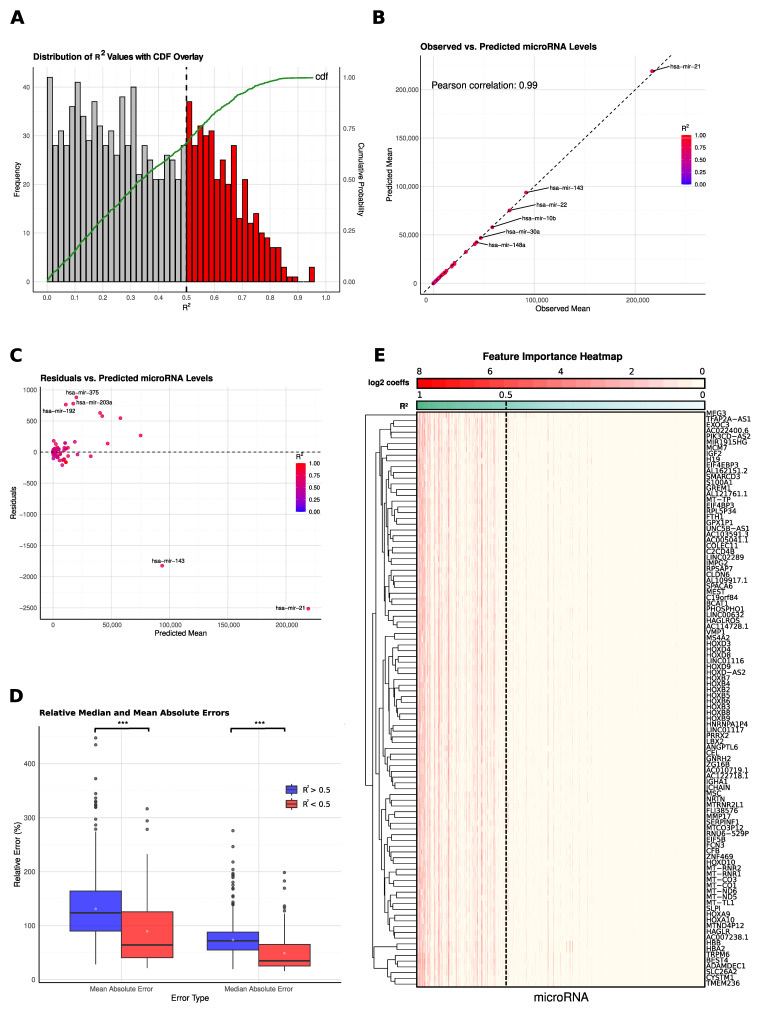
miRNA Prediction Performance and Feature Importance Analysis. (**A**) Distribution of R^2^ values across predicted miRNAs, with a histogram illustrating the range of prediction accuracies and cumulative probability overlayed in green. (**B**) Comparison of observed versus predicted mean miRNA expression levels, shown in a scatterplot with data points colored according to R^2^ values, highlighting prediction accuracy across samples. The strong correlation indicates the model’s effectiveness in estimating overall expression patterns. (**C**) Residual analysis displaying the differences between observed and predicted mean values plotted against predicted mean miRNA levels, with outliers highlighted. This indicates expression ranges where the model performs less consistently. *** denotes *p* < 0.01. (**D**) Boxplots comparing relative errors (median and mean absolute errors) for miRNAs grouped by predicted R^2^ values (<0.5 and >0.5), providing insight into prediction reliability across different accuracy levels. Lower errors in the high-R^2^ group emphasize the model’s robustness for better-predicted miRNAs. (**E**) Clustered heatmap of the top 100 genes with the highest absolute coefficients, showing feature importance by miRNA. Genes are sorted in descending order of R^2^ values, visualizing the predictive contributions across miRNAs, and log2-transformed absolute coefficients are visualized to highlight the relative contribution of each gene across different miRNA models. This panel illustrates the diverse gene contributions underlying miRNA expression and supports the model’s reliance on multiple features.

We thoroughly evaluated the performance of the ridge regression models that we utilized to predict miRNA expression levels from RNA sequencing data, using various statistical metrics. The distribution of R^2^ values across all miRNAs (Figure 1A) reveals a wide range of predictive accuracies. The cumulative distribution function (CDF) overlay shows that 353 out of the 1300 miRNAs analyzed achieve R^2^ values greater than 0.5, demonstrating strong predictive capabilities for these specific targets. This may stem from their inherently high expression levels, as reflected by the median TPM values extracted from respective TCGA samples (R^2^ ≤ 0.5: 0.14; R^2^ > 0.5: 39; see Section 4 for details).

The comparison between observed and predicted miRNA levels (Figure 1B) shows a strong linear correlation, as evidenced by a Pearson correlation coefficient of 0.99, indicating the model’s proficiency in accurately capturing the mean expression levels for the majority of miRNAs, reinforcing the validity of using gene expression data as a reliable surrogate for direct miRNA measurement. In addition to R^2^-based evaluation, we calculated the Spearman correlation between observed and predicted miRNA expression values across all miRNAs. We obtained a mean Spearman correlation of 0.55, indicating a strong monotonic relationship between observed and predicted values.

To evaluate the model’s consistency, we examined the correlation between the coefficients of variation (CV) for observed and predicted miRNA levels. Overall, this correlation was moderate (r = 0.32), indicating some alignment between observed and predicted variability. For miRNAs with R^2^ values above 0.5, the correlation was much stronger (r = 0.98), demonstrating high stability and consistency in predictions. Conversely, for miRNAs with R^2^ values < 0.5, the correlation dropped to 0.20, highlighting greater challenges in accurately predicting these miRNAs. These results reinforce the model’s robustness, especially for miRNAs with higher predictive accuracy.

Residual analysis (Figure 1C) provides additional insights into the model’s robustness and areas for improvement. While the residuals generally cluster around zero, indicating unbiased predictions across most expression levels, certain miRNAs, such as hsa-mir-21, exhibit significant deviations from the trend. These deviations are primarily associated with miRNAs that have very high expression levels, suggesting that outlier values or extreme expression levels may introduce some noise or variability into the predictive model.

The relative errors, both mean absolute error (MAE) and median absolute error (MedAE), were significantly lower for miRNAs with R^2^ > 0.5 compared to those with R^2^ < 0.5, as shown in Figure 1D, with a *p*-value less than 0.01. This highlights the improved predictive accuracy for miRNAs with higher R^2^ values, pointing at the model being more robust for targets with generally higher expression values (Figure 1D).

For the top 100 genes with the highest variability, the feature importance heatmap (Figure 1E) illustrates the absolute log2 values of the coefficients for miRNAs with R^2^ values greater than 0.5, revealing that many features have high coefficients. This indicates that the prediction of miRNA expression is not driven by a single gene but rather by a complex interaction among multiple genes. The presence of several genes with substantial coefficients underscores the multifactorial nature of miRNA regulation, validating the model’s strategy of using a diverse set of gene expression data to enhance predictive accuracy. Conversely, for miRNAs with R^2^ values < 0.5, there are no significantly high coefficients, suggesting a lack of strong predictive features and highlighting the challenges in predicting these miRNAs accurately (Figure 1E).

To assess whether alternative modeling approaches could improve predictive accuracy, we also implemented Lasso regression as a linear model and Random Forest regression as a non-linear model. Both approaches underperformed relative to ridge regression, with 196 miRNAs (Lasso) and 239 miRNAs (Random Forest) achieving R^2^ values > 0.5. A full comparison of model performance metrics is provided in Supplementary Tables S1–S3.

Overall, the results demonstrate that our ridge regression models provide a robust framework for predicting miRNA expression from RNA-seq data, particularly for miRNAs with clear expression patterns.

### 2.2. MiRNA–Gene Network Connectivity and Centrality Analysis

**Figure 2:**
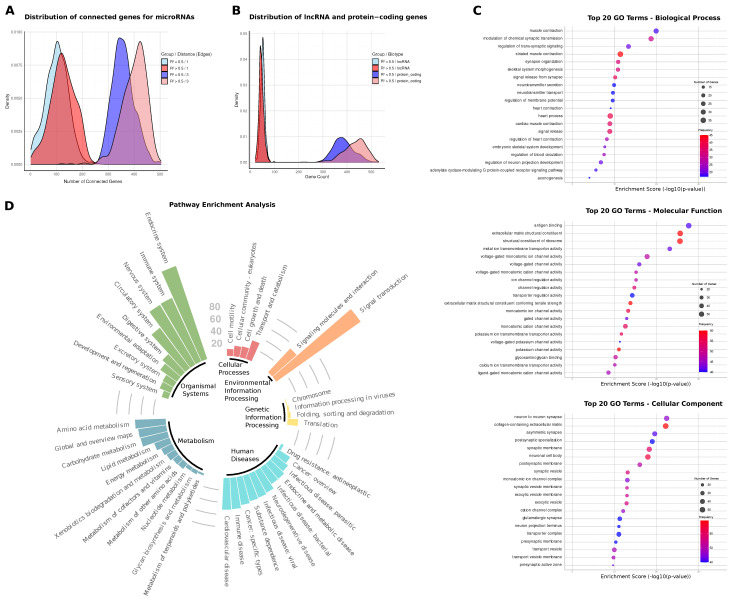
Functional Characterization of Predictive Genes in miRNA Networks. (**A**) Distribution of direct and 3-node distance gene interactions among the top 632 predictive genes for each miRNA, divided into two groups: R^2^ > 0.5 and R^2^ < 0.5. Distribution plot highlights differences in connectivity for high and low-accuracy miRNAs. Higher connectivity in the well-predicted group reflects stronger and more direct regulatory relationships in these miRNA models. (**B**) Density distribution of gene biotypes, with long non-coding RNAs and protein-coding genes represented among the top predictive genes for each miRNA, split by R^2^ category (R^2^ > 0.5 and R^2^ < 0.5). Protein-coding genes dominate predictive gene sets, particularly for miRNAs with high prediction accuracy. (**C**) Gene Ontology (GO) term analysis results for the top 20 enriched terms in biological process (BP), cellular component (CC), and molecular function (MF) categories. Enrichment is based on the frequency of significance across miRNAs, using the top 632 predictive genes per miRNA (see Supplementary Table S5). (**D**) KEGG-pathway enrichment analysis for the top predictive genes across miRNAs, illustrated in a bar plot where the height of each bar represents the number of miRNAs significantly enriched in each pathway. This visualization highlights the pathways most frequently associated with genes that predict miRNA expression.

We analyzed the connectivity of the top 3% (632) of predictive genes, determined by the highest absolute coefficients for each miRNA, within the gene–miRNA network (Figure 2A). For miRNAs with R^2^ > 0.5, a higher proportion of predictive genes were found to be directly interacting with the miRNA (1-node distance), averaging 125 genes, compared to 102 genes for miRNAs with R^2^ < 0.5. Additionally, when examining the 3-node distance (3 degrees of separation), the difference between the two groups becomes more pronounced, with miRNAs that are better predicted (R^2^ > 0.5) showing an average of 401 connected genes, compared to 358 for those with R^2^ < 0.5. This suggests that miRNAs with higher predictive power tend to form stronger direct regulatory relationships with their target genes, highlighting the connection between prediction accuracy and regulatory interactions (Figure 2A).

We also examined the distribution of lncRNAs and protein-coding genes among the top predictive genes. Both groups, miRNAs with R^2^ > 0.5 and R^2^ < 0.5, had a similarly small proportion of lncRNAs among the top predictive genes. However, the proportion of protein-coding genes was higher for miRNAs with R^2^ > 0.5 (Figure 2B).

Analysis of the network’s communities (groups of densely connected nodes, see Methods for details) shows variability in the proportion of well-predicted miRNAs (R^2^ > 0.5) across different communities, with some communities having a higher concentration of accurately predicted miRNAs. While this observation suggests differences in the predictive relationships within these communities, no consistent pattern was observed regarding community size (nodes/edges) and prediction quality (see Supplementary Table S4).

We analyzed the relationship between miRNA expression variability and their connectivity within the network by focusing on the 55 miRNAs with a high coefficient of variation (CV > 10). Correlating their R^2^ values with different network centrality measures revealed notable relationships: a Pearson correlation of 0.49 for both degree centrality and betweenness centrality and 0.47 for eigenvector centrality. These positive correlations suggest that miRNAs with higher variability in expression tend to be predicted better when they occupy more central and influential positions in the network.

This finding implies that miRNAs with significant network connectivity—either by having numerous direct interactions (degree centrality), being central to communication pathways (betweenness centrality), or influencing other highly connected nodes (eigenvector centrality)—are more likely to exhibit predictable expression patterns. This could indicate that miRNAs deeply embedded in the regulatory network play crucial roles in maintaining network stability, which could explain why their expression is better captured by predictive models.

### 2.3. Biological Signatures of Predictive miRNA Regulators

In the GO term analysis of the top predictive genes for miRNAs with R^2^ > 0.5, we found that many terms in the biological process category are related to synaptic function and cardiovascular processes. Notably, terms such as the modulation of chemical synaptic transmission, synapse organization, neurotransmitter secretion, and the regulation of neuron projection development had the highest number of predictive genes associated with them. In addition, cardiovascular-related terms like cardiac muscle contraction, heart process, and the regulation of blood circulation were also highly enriched, underscoring the involvement of these genes in critical physiological pathways (Figure 2C).

In the molecular function category, many of the enriched terms pertained to ion channel activity, particularly those involved in synaptic signaling. Higher-level categories like voltage-gated ion channel activity, monoatomic cation channel activity, and potassium channel activity dominated, with a large number of genes contributing to these functions. This suggests that most well-predicted miRNA families have predictive genes involved in regulating ion transport and signaling, further emphasizing their role in synaptic function and neuronal regulation (Figure 2C).

The cellular component category also reflected a strong focus on synaptic structures, with terms such as synaptic vesicle membrane, postsynaptic membrane, and neuronal cell body being the most enriched. These terms highlight the cellular environments where the predictive genes are most active, particularly in synapse-related functions. The enrichment in these synaptic components suggests that the genes associated with better-predicted miRNAs are often localized to critical regions involved in neural communication (Figure 2C).

These findings illustrate that the majority of well-predicted miRNA families have predictive genes that are heavily involved in synaptic and cardiovascular processes, as reflected by their enrichment in both functional and structural terms across the GO categories.

### 2.4. miRNA-Linked Pathway Enrichments

We subsequently performed pathway enrichment analysis using the KEGG database for the same set of predictive genes. Pathways significantly enriched across the majority of miRNAs include signal transduction, which involved 170 out of the 353 miRNAs considered (48%), the endocrine system with 160 miRNAs (45%), the nervous system with 113 miRNAs (32%), and cardiovascular diseases with 52 miRNAs (15%). These findings align with the GO term enrichment results, emphasizing synaptic and cardiovascular processes (Figure 2D).

**Figure 3:**
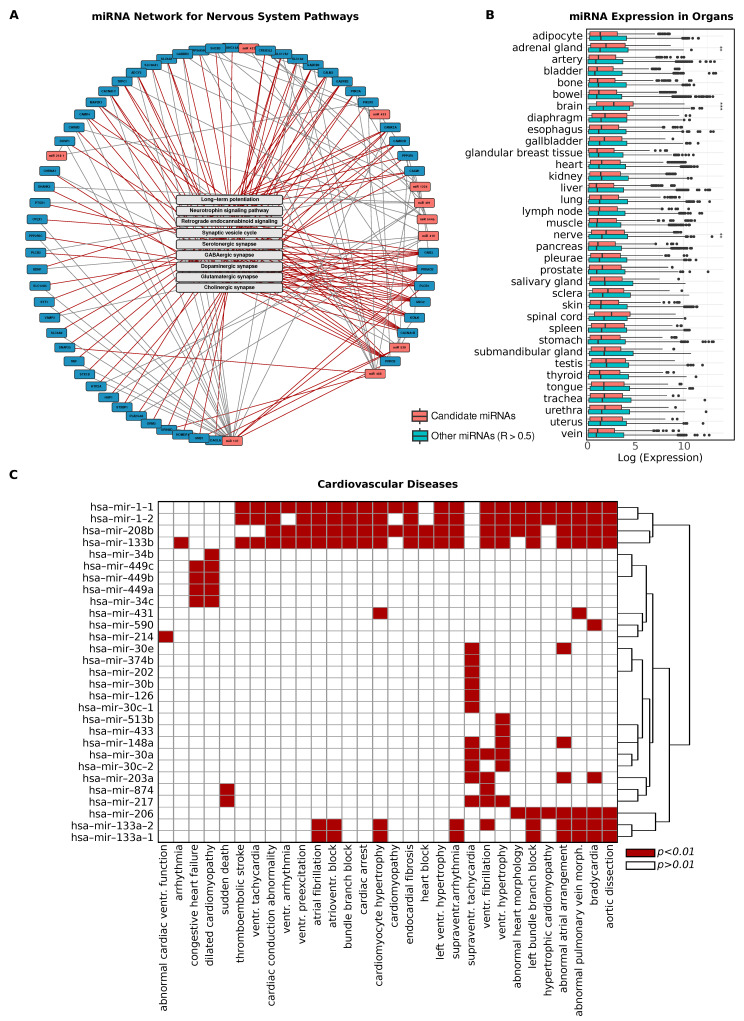
Predictive miRNAs in Neural and Cardiovascular Pathways. (**A**) Filtered network of miRNA-gene interactions, focused on pathways related to the nervous system. Network visualizes specific interactions in nervous system-associated pathways, emphasizing miRNAs like miR-137 that show high connectivity. Red edges indicate interactions involving genetic predictors and direct pathway associations, while grey lines represent additional connections with non-predictive regulators and indirect pathway associations. (**B**) Bar plot displaying tissue-specific expression levels of miRNAs that are filtered for signal transduction pathways and for the background of all other miRNAs predicted with R^2^ > 0.5. Elevated expression in neural tissues supports the functional relevance of the filtered miRNA set; *** denotes *p* < 0.01, ** denotes *p* between 0.01 and 0.05. (**C**) Heatmap showing miRNAs significantly enriched in cardiovascular disease pathways (*p* < 0.01), linking predictive genes to disease-relevant regulatory modules.

We then filtered the miRNA–gene network to focus specifically on the genes associated with the pathways identified in the previous enrichment analysis, retaining only miRNAs directly connected to these genes. Additionally, we incorporated specific pathways corresponding to the enriched terms. For the nervous system, we presented this filtered network in Figure 3A, where key miRNAs such as miR-137 and miR-488 emerged as highly connected nodes within the network. This strategy resulted in the selection of 11 miRNAs, revealing a clear concentration of regulatory interactions within neural-associated pathways.

We applied the same filtering strategy to isolate pathways associated with signal transduction, which also led to the selection of 43 miRNAs. For these miRNAs, we further analyzed their tissue-specific expression patterns using the TAM 2.0 database [29]. We contrasted their expression levels with the expression profiles of all other miRNAs with R^2^ > 0.5. Through this comparison, we identified significantly higher normalized expression levels of these signal-transduction-associated miRNAs in several key tissues, particularly the brain, nerve, and adrenal gland (Figure 3B).

These findings are consistent with the biological roles of signal transduction and nervous system pathways and provide additional validation of our network filtering methodology. The enriched expression in neural and endocrine-related tissues supports the functional relevance of the extracted miRNAs and highlights their potential regulatory impact within these critical systems. This underscores the biological coherence of our approach, linking the predictive genes and pathways to specific tissue contexts, thus reinforcing the importance of these miRNAs in neural and signal-transduction-related processes.

### 2.5. Cardiovascular Disease Associations in Predictive Gene Networks

We next conducted disease enrichment analysis for the predictive genes of miRNAs with R^2^ > 0.5, focusing on terms related to cardiovascular diseases, which were among the most prevalent in the pathway enrichment results (Figure 3C). Notably, the terms arrhythmia, abnormal cardiac ventricular function, and cardiac conduction abnormality were among the most frequent. Specifically, we observed significant enrichment of cardiovascular-disease-related terms for the predictive genes of miR-1-1, miR-1-2, miR-208b, and miR-133b, indicating their strong association with various cardiovascular conditions. miR-208b and miR-133b also appeared consistently when constructing the subnetwork for cardiovascular diseases, which included a total of 16 miRNAs. This reinforces the role of these specific miRNAs in cardiovascular regulation and highlights their potential importance in disease-associated regulatory networks.

## 3. Discussion

In this study, we employed ridge regression to predict miRNA expression from RNA sequencing data, leveraging its strengths in handling high-dimensional data and capturing multicollinearity. Ridge regression has been widely used in genetic studies to address the challenges posed by complex datasets, particularly those involving gene regulatory networks [30,31,32,33]. Its ability to manage large numbers of correlated features while maintaining robust predictions makes it ideal for exploring the regulatory interactions between miRNAs and their target genes, which are often characterized by overlapping regulatory roles and multicollinear gene expression profiles [11,34,35]. Our models performed well for over 353 miRNAs (R^2^ > 0.5), likely due to their higher expression levels facilitating stronger signal detection.

Among the top 100 miRNAs with the highest mean expression, only eight were predicted with R^2^ values below 0.5, reinforcing the strength of the model in capturing the regulatory dynamics of highly expressed miRNAs.

In contrast, miRNAs with lower expression levels posed a greater challenge. This is likely due to their lower signal-to-noise ratios, making it difficult for the model to distinguish true signals from background noise. Random Forest regression did not improve predictions, suggesting limited predictability may stem from biological variability or sparse input features. Lastly, regularization in ridge regression tends to shrink the coefficients of low-expression miRNAs, reducing their predictive accuracy. However, this trade-off is crucial for preventing overfitting as the model must balance capturing meaningful patterns without allowing noise to dominate the predictions [36,37,38]. A critical factor in the model’s success was carefully selecting the regularization parameter (alpha), which we set to 11,000. This relatively high regularization was essential due to the inclusion of a large number of gene features.

While existing methods predominantly address miRNA expression imputation through techniques such as constrained least squares and GO-based similarity measures, our approach broadens the application to both healthy and tumor tissues, enhancing predictive performance without the need for imputation strategies [20,39]. Although no current tool provides pretrained models for direct miRNA prediction from gene expression, we benchmark our approach against miRSCAPE [22], which infers miRNA levels from scRNA-seq and reports a mean Spearman correlation of 0.45 across 10 TCGA cancer cohorts, focusing on miRNAs expressed in over 50% of samples. miRSCAPE also reports improved performance over miREACT based on bulk RNA-seq data [21]. In comparison, our model achieves a higher mean Spearman correlation of 0.55 across 1300 miRNAs, despite using a less stringent filtering threshold. We attribute this performance gain to two factors: the use of a linear ridge regression model, which generalizes well in high-dimensional settings, and the approximately two-fold increase in training data as we aggregated samples across all cancer types rather than limiting to individual cohorts. By predicting miRNA expression directly from RNA-seq-derived expression matrices, our set of models offers broader applicability without the need for pre-existing miRNA profiles. Our study is further strengthened by the availability of scripts that enable researchers to train their own models using TCGA or similar datasets, providing flexibility in adapting the approach to diverse research questions. By integrating these computational tools, we aim to facilitate reproducibility and extend the practical applications of miRNA prediction. These resources empower users to not only predict miRNA expression but also explore novel regulatory relationships tailored to specific datasets and biological systems.

Our analysis of connectivity within the miRNA–gene network reveals significant relationships between prediction accuracy (R^2^ > 0.5) and the degree of direct gene interactions. Positive correlations with degree centrality and betweenness centrality suggest that miRNAs with greater regulatory influence tend to exhibit more stable and predictable expression patterns. This highlights the importance of considering miRNAs not in isolation but within the context of their broader network interactions. miRNAs with high centrality likely act as regulatory hubs, influencing a wide range of target genes across critical biological pathways.

We observed a significant enrichment of biological processes related to synaptic function and cardiovascular systems. Terms such as “synapse organization” and “cardiac muscle contraction” consistently appeared in the GO analysis for miRNAs with high R^2^ values, indicating a role in crucial physiological pathways. This is further validated by the pathway enrichment results, where signal transduction and cardiovascular pathways dominated. Filtered miRNAs, such as miR-1-1, miR-208b, and miR-133b, which showed strong associations with cardiovascular-disease-related pathways, have been extensively documented as key players in their role in cardiovascular disease progression and biomarkers in the literature [40,41,42,43], providing further validation of our findings and highlighting their critical role in cardiovascular regulation. miRNAs may play an essential role in the heart’s adaptability to varying physiological stimuli, allowing for rapid regulatory responses critical in maintaining cardiac rhythm and contractility [44,45].

In neurons, miRNAs may function as highly localized regulatory elements, helping to control mRNA pools in distant cellular locations like dendrites. This setup supports rapid, synapse-specific protein synthesis and is well suited to the nervous system’s dynamic demands, where miRNAs act as localized regulators that inhibit protein production from stored mRNAs [46]. This regulatory mechanism is consistent with the presence of multiple polyadenylation sites in neural transcripts [47,48], allowing for flexible transcript pools finely regulated by miRNAs.

By subsetting these miRNA–gene networks based on genes predictive for miRNAs and enriched in signal transduction pathways, we identified miRNAs specifically linked to these pathways. When cross-referenced with independent databases, these selected miRNAs also showed higher expression in their respective tissues that are nerve, adrenal gland, and brain.

This organ-specific expression additionally validates our methodological approach, indicating that the miRNAs identified as key players especially in synaptic and cardiovascular pathways are biologically relevant. This finding is consistent with existing research that demonstrates the tissue-specific roles of miRNAs in both the heart and brain [49,50]. Our analysis highlights several miRNAs—miR-1, miR-133b, miR-208b, miR-137, and miR-124—that not only show high predictive accuracy but are also strongly linked to known biological functions, reinforcing the validity of our models. miR-1 and miR-133b are well-established regulators of cardiac function, implicated in muscle differentiation, arrhythmias, and heart failure [51,52]. miR-208b, intronic to MYH7, is known to regulate cardiac hypertrophy [53]. In the nervous system, miR-137 plays a central role in regulating synaptic vesicle trafficking, neurotransmitter release, and presynaptic plasticity—functions essential for proper synaptic activity and neuronal signaling [54]. miR-124, one of the most abundant brain miRNAs, has been shown to fine-tune the timing and amount of adult neurogenesis [55]. The strong model performance and pathway enrichment of these miRNAs support the biological coherence of our predictions and functional connection to respective genetic regulators.

One limitation of this study is the model’s reduced accuracy in predicting miRNAs with low expression levels, likely due to weaker signal-to-noise ratios and sparse data for these miRNAs. Additionally, the miRNA–gene network used in our analysis is based on current datasets, which are continuously evolving as new experimental data becomes available. As a result, some regulatory interactions may be missing, and the network may not fully capture all relevant biological relationships, potentially limiting the scope and applicability of our findings.

This study illustrates the effectiveness of ridge regression for predicting miRNA expression levels from gene expression data, offering a computational alternative to direct miRNA measurement. By analyzing the predictive gene–miRNA relationships, we revealed key insights into the functional roles of miRNAs, particularly in synaptic and cardiovascular processes. Our findings provide a foundation for further exploration of miRNA regulation in disease contexts, and the framework developed here has the potential for broader applications in miRNA-related research. In future work, integrating deep-learning-based approaches may offer improved adaptability and performance, particularly for harmonizing and modeling data originating from multiple experimental platforms [56].

## 4. Methods

### 4.1. Data Collection

We sourced expression data from TCGA [28], selecting all samples across all cancer types that included both miRNA sequencing and RNA-seq data from the same tissue condition (either tumor or normal). Our dataset comprised 10,464 matched expression profiles, with 9828 derived from tumor tissues and 636 from normal tissues. To ensure uniformity and comparability across all samples, we used normalized expression values using Transcripts Per Million (TPMs). This normalization method accounts for differences in sequencing depth and gene length, providing a standardized framework for subsequent analysis.

### 4.2. Data Preparation

To model miRNA expression levels, we utilized gene expression values as predictive features. The initial data preparation phase involved several key steps: loading the RNA-seq and miRNA-seq datasets and filtering the feature matrix by removing rows with more than 1050 zeros and 1050 NaNs, a threshold set to 10% of the total samples. This threshold was selected to eliminate features with excessive missing or non-expressed values while retaining a broad set of informative predictors. For the label matrix, we applied a less stringent threshold of 10,000 zeros and 10,000 NaNs. Given that miRNA expression is often tissue-specific and generally lower in abundance, this threshold allows us to retain miRNAs present in at least 325 samples, capturing approximately 70% of miRNAs, even those expressed at lower levels. This filtering strategy enabled elimination of non-informative features and labels, enhancing the robustness of subsequent analyses. We also removed features with zero variance to focus on informative predictors.

Next, we applied a Z-transformation using the *StandardScaler* function from the scikit-learn library [57] to standardize the features so that the values of different features are on the same scale to ensure that the regularization applies equally to all coefficients, which is important for the performance of the ridge regression.

### 4.3. miRNA Expression Modeling

For each miRNA, the dataset was split into training (80%) and test (20%) subsets using random sampling, and ridge regression was applied using the *Ridge* function from scikit-learn. This regularization technique is effective in managing multicollinearity and high-dimensional datasets, chosen for its ability to prevent overfitting and enhance prediction stability. The regularization parameter (alpha) was set at 11,000 after initial tuning after an initial grid search with 5-fold cross-validation on the same training set over a range of values (1, 10, 100, 1000, 10,000, 11,000, and 15,000), selecting 11,000 as it yielded the highest number of miRNAs with R^2^ values greater than 0.5 while maintaining the lowest mean absolute error. This methodology ensures that the test set remained completely independent throughout the training and model optimization process to provide an unbiased evaluation of model performance.

Interestingly, running the same model with the top 3% of features with the highest absolute coefficients yielded similar results (Supplementary Tables S1 and S2). While we focused on these top features for downstream analysis, we retained all features for modeling, as computing efficiency was not significantly impacted, and the overall results improved. Given the set of TPM-normalized gene expression data, our method can be applied to predict miRNA expression using the provided predict_microRNA_expression.py script.

### 4.4. Model Performance Evaluation

The models were trained on the training set and evaluated on the test set using metrics such as R^2^, median absolute error, and mean absolute error (Supplementary Tables S1 and S2). These metrics provided a comprehensive view of model performance across different miRNAs. The final dataset consisted of 21,044 features (mRNA expression data) and 1300 miRNAs as targets. All features were retained in the final models as feature exclusion did not improve model efficiency and retaining them enhanced predictive accuracy. The top 3% genes with the highest absolute coefficients for miRNAs predicted with R^2^ > 0.5 are provided in Supplementary Table S6. The entire modeling process was conducted using Python version 3.10 and R version 4.4. For comparison, we also trained models using the *Lasso* and *RandomForestRegressor* functions from the scikit-learn library [57], using the same training and test splits as for ridge regression (Supplementary Table S3).

### 4.5. Network Construction and Subsetting

Following the predictive modeling, we constructed a network of miRNAs and their target genes. This network was built by combining experimentally validated interactions from the miRTarBase [34], DIANA-TarBase [35], and TRANSFAC databases [58]. Additionally, we complemented these interactions with predicted conserved interactions from the TargetScanHuman 8.0 [9] with conservation scores higher than 0.5 to ensure a comprehensive representation of miRNA–gene interactions. In this network, each node represents a gene or miRNA, and the edges represent the interactions between them.

### 4.6. Connectivity Metrics

To analyze miRNA connectivity within the network, we used the biomaRt package [59] to classify gene biotypes as protein-coding or long non-coding RNAs (lncRNAs). Key connectivity metrics were computed using the igraph package [60]: *degree* assessed the number of direct interactions, *betweenness* measured the role of miRNAs as bridges in the network, and *evcent* evaluated their influence based on connections. Communities were computed with *cluster_louvain.* Pearson correlation coefficients between R^2^ values and centrality measures were calculated using the *cor* function. Network metrics for miRNA–gene regulatory interactions, including connectivity measures, centrality scores, and community structure, are provided in Supplementary Tables S4 and S7.

### 4.7. Gene Ontology (GO) Term Analysis

For the Gene Ontology (GO) term analysis, we focused on the top 3% of the most predictive genes for each miRNA, selected based on R^2^ values greater than 0.5. This analysis concentrated on significant findings with adjusted *p*-values smaller than 0.05. Utilizing the *enrichGO* function from clusterProfiler [61], we examined biological processes (BP), molecular functions (MF), and cellular components (CC) to uncover the biological and molecular underpinnings influenced by these highly predictive genes. Detailed GO term enrichment results are provided in Supplementary Table S5.

### 4.8. Pathway Analysis

For the pathway analysis, we again focused on the top 3% most predictive genes for each miRNA with R^2^ values greater than 0.5. We performed enrichment analysis using the KEGG database [62], applying a threshold of adjusted *p*-values smaller than 0.05 to identify significant pathways (see Supplementary Tables S8 and S9). This involved utilizing the *enrichKEGG* function from the clusterProfiler [61] package to map the predictive genes to their associated biological pathways, providing insights into the regulatory frameworks governing miRNA-mediated gene expression.

### 4.9. Disease Enrichment

The disease enrichment analysis was conducted using the top 3% of predictive genes for each miRNA, identified based on R^2^ values greater than 0.5. These genes were cross-referenced with the Human Phenotype Ontology database [63] through the g:Profiler web platform [64], with a *p*-value cutoff of 0.01 (see Supplementary Table S10).

### 4.10. Organ-Specific miRNA Expression

For the comparison of organ-specific miRNA expression levels between miRNAs associated with signal transduction pathways and all other miRNAs with R^2^ values greater than 0.5, we utilized the TAM 2.0 database [29] to extract miRNA levels for each organ. The expression levels of the pathway-specific miRNAs were contrasted with the remaining miRNAs, and the significance of the differences was assessed using a *t*-test using *t* test function of the R stats library.

## 5. Conclusions

We present a robust ridge regression framework for predicting miRNA expression from RNA-seq data, identifying over 350 miRNAs with high predictive accuracy. Our models uncover key gene–miRNA regulatory relationships, particularly in synaptic and cardiovascular pathways, and highlight tissue-specific expression patterns linked to biological function and disease. The accompanying pretrained models and scripts offer a reproducible tool for miRNA prediction, with broad applicability in both research and clinical settings.

## Data Availability

The dataset supporting the conclusions of this article is included within the article and its additional Supplementary Tables. The scripts for predicting miRNA expression from gene expression data and for training custom models are available at https://github.com/mcihan0bioinf/microRNA_prediction, accessed on 6 June 2025. Supplementary Tables include: comprehensive benchmarking results for all features (S1) and the top 3% predictive features (S2); results for alternative machine learning models (S3); community membership of microRNAs within the network (S4); results of gene ontology enrichment (S5); a list of genetic predictors of miRNAs (S6); network metrics (S7); KEGG pathway enrichment analysis (S8); identified microRNA pathways (S9); disease association findings (S10).

## Supplementary Materials

The following supporting information can be downloaded at https://www.mdpi.com/article/10.3390/ijms26125757/s1.

## References

[1] Bartel D.P. (2009). MicroRNAs: Target Recognition and Regulatory Functions. Cell.

[2] Hayes J., Peruzzi P.P., Lawler S. (2014). MicroRNAs in Cancer: Biomarkers, Functions and Therapy. Trends Mol. Med..

[3] Kozomara A., Griffiths-Jones S. (2014). miRBase: Annotating High Confidence microRNAs Using Deep Sequencing Data. Nucleic Acids Res..

[4] Stempor P.A., Cauchi M., Wilson P. (2012). MMpred: Functional miRNA—mRNA Interaction Analyses by miRNA Expression Prediction. BMC Genom..

[5] Huang J.C., Babak T., Corson T.W., Chua G., Khan S., Gallie B.L., Hughes T.R., Blencowe B.J., Frey B.J., Morris Q.D. (2007). Using Expression Profiling Data to Identify Human microRNA Targets. Nat. Methods.

[6] Ruike Y., Ichimura A., Tsuchiya S., Shimizu K., Kunimoto R., Okuno Y., Tsujimoto G. (2008). Global Correlation Analysis for Micro-RNA and mRNA Expression Profiles in Human Cell Lines. J. Hum. Genet..

[7] Alevizos I., Illei G.G. (2010). MicroRNAs as Biomarkers in Rheumatic Diseases. Nat. Rev. Rheumatol..

[8] Reda El Sayed S., Cristante J., Guyon L., Denis J., Chabre O., Cherradi N. (2021). MicroRNA Therapeutics in Cancer: Current Advances and Challenges. Cancers.

[9] McGeary S.E., Lin K.S., Shi C.Y., Pham T.M., Bisaria N., Kelley G.M., Bartel D.P. (2019). The Biochemical Basis of microRNA Targeting Efficacy. Science.

[10] Jin S., Zeng X., Fang J., Lin J., Chan S.Y., Erzurum S.C., Cheng F. (2019). A Network-Based Approach to Uncover microRNA-Mediated Disease Comorbidities and Potential Pathobiological Implications. npj Syst. Biol. Appl..

[11] Cihan M., Andrade-Navarro M.A. (2022). Detection of Features Predictive of microRNA Targets by Integration of Network Data. PLoS ONE.

[12] van Iterson M., Bervoets S., de Meijer E.J., Buermans H.P., ‘t Hoen P.A.C., Menezes R.X., Boer J.M. (2013). Integrated Analysis of microRNA and mRNA Expression: Adding Biological Significance to microRNA Target Predictions. Nucleic Acids Res..

[13] Xuan J., Shi L., Guo L. (2013). microRNA Profiling: Strategies and Challenges. microRNAs in Toxicology and Medicine.

[14] Wright C., Rajpurohit A., Burke E.E., Williams C., Collado-Torres L., Kimos M., Brandon N.J., Cross A.J., Jaffe A.E., Weinberger D.R. (2019). Comprehensive Assessment of Multiple Biases in Small RNA Sequencing Reveals Significant Differences in the Performance of Widely Used Methods. BMC Genom..

[15] Benesova S., Kubista M., Valihrach L. (2021). Small RNA-Sequencing: Approaches and Considerations for miRNA Analysis. Diagnostics.

[16] Matullo G., Naccarati A., Pardini B. (2016). MicroRNA Expression Profiling in Bladder Cancer: The Challenge of next-Generation Sequencing in Tissues and Biofluids. Int. J. Cancer.

[17] Backes C., Sedaghat-Hamedani F., Frese K., Hart M., Ludwig N., Meder B., Meese E., Keller A. (2016). Bias in High-Throughput Analysis of miRNAs and Implications for Biomarker Studies. Anal. Chem..

[18] Madadi S., Schwarzenbach H., Lorenzen J., Soleimani M. (2019). MicroRNA Expression Studies: Challenge of Selecting Reliable Reference Controls for Data Normalization. Cell. Mol. Life Sci..

[19] Schwarzenbach H., da Silva A.M., Calin G., Pantel K. (2015). Data Normalization Strategies for MicroRNA Quantification. Clin. Chem..

[20] Webber J.W., Elias K.M. (2022). Fast and Robust Imputation for miRNA Expression Data Using Constrained Least Squares. BMC Bioinform..

[21] Nielsen M.M., Pedersen J.S. (2021). miRNA Activity Inferred from Single Cell mRNA Expression. Sci. Rep..

[22] Olgun G., Gopalan V., Hannenhalli S. (2022). miRSCAPE—Inferring miRNA Expression from scRNA-Seq Data. iScience.

[23] Cheng C., Li L.M. (2008). Inferring MicroRNA Activities by Combining Gene Expression with MicroRNA Target Prediction. PLoS ONE.

[24] Le T.D., Liu L., Tsykin A., Goodall G.J., Liu B., Sun B.-Y., Li J. (2013). Inferring microRNA–mRNA Causal Regulatory Relationships from Expression Data. Bioinformatics.

[25] Tan H., Huang S., Zhang Z., Qian X., Sun P., Zhou X. (2019). Pan-Cancer Analysis on microRNA-Associated Gene Activation. EBioMedicine.

[26] Monteys A.M., Spengler R.M., Wan J., Tecedor L., Lennox K.A., Xing Y., Davidson B.L. (2010). Structure and Activity of Putative Intronic miRNA Promoters. RNA.

[27] Hoerl A.E., Kennard R.W. (1970). Ridge Regression: Biased Estimation for Nonorthogonal Problems. Technometrics.

[28] Weinstein J.N., Collisson E.A., Mills G.B., Shaw K.R.M., Ozenberger B.A., Ellrott K., Shmulevich I., Sander C., Stuart J.M., Cancer Genome Atlas Research Network (2013). The Cancer Genome Atlas Pan-Cancer Analysis Project. Nat. Genet..

[29] Li J., Han X., Wan Y., Zhang S., Zhao Y., Fan R., Cui Q., Zhou Y. (2018). TAM 2.0: Tool for MicroRNA Set Analysis. Nucleic Acids Res..

[30] Frouin A., Dandine-Roulland C., Pierre-Jean M., Deleuze J.-F., Ambroise C., Le Floch E. (2020). Exploring the Link Between Additive Heritability and Prediction Accuracy from a Ridge Regression Perspective. Front. Genet..

[31] Novianti P.W., Snoek B.C., Wilting S.M., van de Wiel M.A. (2017). Better Diagnostic Signatures from RNAseq Data through Use of Auxiliary Co-Data. Bioinformatics.

[32] Mbatchou J., Barnard L., Backman J., Marcketta A., Kosmicki J.A., Ziyatdinov A., Benner C., O’Dushlaine C., Barber M., Boutkov B. (2021). Computationally Efficient Whole-Genome Regression for Quantitative and Binary Traits. Nat. Genet..

[33] Liu C., Wei D., Xiang J., Ren F., Huang L., Lang J., Tian G., Li Y., Yang J. (2020). An Improved Anticancer Drug-Response Prediction Based on an Ensemble Method Integrating Matrix Completion and Ridge Regression. Mol. Ther.—Nucleic Acids.

[34] Huang H.-Y., Lin Y.-C.-D., Cui S., Huang Y., Tang Y., Xu J., Bao J., Li Y., Wen J., Zuo H. (2022). miRTarBase Update 2022: An Informative Resource for Experimentally Validated miRNA-Target Interactions. Nucleic Acids Res..

[35] Karagkouni D., Paraskevopoulou M.D., Chatzopoulos S., Vlachos I.S., Tastsoglou S., Kanellos I., Papadimitriou D., Kavakiotis I., Maniou S., Skoufos G. (2018). DIANA-TarBase v8: A Decade-Long Collection of Experimentally Supported miRNA-Gene Interactions. Nucleic Acids Res..

[36] McDonald G.C. (2009). Ridge Regression. WIREs Comput. Stat..

[37] Cule E., De Iorio M. (2013). Ridge Regression in Prediction Problems: Automatic Choice of the Ridge Parameter. Genet. Epidemiol..

[38] Zhang R., McDonald G.C. (2005). Characterization of Ridge Trace Behavior. Commun. Stat.—Theory Methods.

[39] Yang Y., Xu Z., Song D. (2016). Missing Value Imputation for microRNA Expression Data by Using a GO-Based Similarity Measure. BMC Bioinform..

[40] Chistiakov D.A., Orekhov A.N., Bobryshev Y.V. (2016). Cardiac-Specific miRNA in Cardiogenesis, Heart Function, and Cardiac Pathology (with Focus on Myocardial Infarction). J. Mol. Cell. Cardiol..

[41] Navickas R., Gal D., Laucevičius A., Taparauskaitė A., Zdanytė M., Holvoet P. (2016). Identifying Circulating microRNAs as Biomarkers of Cardiovascular Disease: A Systematic Review. Cardiovasc. Res..

[42] Kaur A., Mackin S.T., Schlosser K., Wong F.L., Elharram M., Delles C., Stewart D.J., Dayan N., Landry T., Pilote L. (2020). Systematic Review of microRNA Biomarkers in Acute Coronary Syndrome and Stable Coronary Artery Disease. Cardiovasc. Res..

[43] Widera C., Gupta S.K., Lorenzen J.M., Bang C., Bauersachs J., Bethmann K., Kempf T., Wollert K.C., Thum T. (2011). Diagnostic and Prognostic Impact of Six Circulating microRNAs in Acute Coronary Syndrome. J. Mol. Cell. Cardiol..

[44] Small E.M., Olson E.N. (2011). Pervasive Roles of microRNAs in Cardiovascular Biology. Nature.

[45] Kalozoumi G., Yacoub M., Sanoudou D. (2014). MicroRNAs in Heart Failure: Small Molecules with Major Impact. Glob. Cardiol. Sci. Pract..

[46] Sambandan S., Akbalik G., Kochen L., Rinne J., Kahlstatt J., Glock C., Tushev G., Alvarez-Castelao B., Heckel A., Schuman E.M. (2017). Activity-Dependent Spatially Localized miRNA Maturation in Neuronal Dendrites. Science.

[47] Liu X., Xie H., Liu W., Zuo J., Li S., Tian Y., Zhao J., Bai M., Li J., Bao L. (2024). Dynamic Regulation of Alternative Polyadenylation by PQBP1 during Neurogenesis. Cell Rep..

[48] Cihan M., Schmauck G., Sprang M., Andrade-Navarro M.A. (2025). Unveiling cell-type-specific microRNA networks through alternative polyadenylation in glioblastoma. BMC Biol..

[49] Lagos-Quintana M., Rauhut R., Yalcin A., Meyer J., Lendeckel W., Tuschl T. (2002). Identification of Tissue-Specific MicroRNAs from Mouse. Curr. Biol..

[50] Ludwig N., Leidinger P., Becker K., Backes C., Fehlmann T., Pallasch C., Rheinheimer S., Meder B., Stähler C., Meese E. (2016). Distribution of miRNA Expression across Human Tissues. Nucleic Acids Res..

[51] Zhao Y., Ransom J.F., Li A., Vedantham V., von Drehle M., Muth A.N., Tsuchihashi T., McManus M.T., Schwartz R.J., Srivastava D. (2007). Dysregulation of Cardiogenesis, Cardiac Conduction, and Cell Cycle in Mice Lacking miRNA-1-2. Cell.

[52] Yang B., Lin H., Xiao J., Lu Y., Luo X., Li B., Zhang Y., Xu C., Bai Y., Wang H. (2007). The Muscle-Specific microRNA miR-1 Regulates Cardiac Arrhythmogenic Potential by Targeting GJA1 and KCNJ2. Nat. Med..

[53] van Rooij E., Sutherland L.B., Qi X., Richardson J.A., Hill J., Olson E.N. (2007). Control of Stress-Dependent Cardiac Growth and Gene Expression by a MicroRNA. Science.

[54] Mahmoudi E., Cairns M.J. (2017). MiR-137: An Important Player in Neural Development and Neoplastic Transformation. Mol. Psychiatry.

[55] Sun J., Sun J., Ming G., Song H. (2011). Epigenetic Regulation of Neurogenesis in the Adult Mammalian Brain. Eur. J. Neurosci..

[56] Chen F., Wang Y.-C., Wang B., Kuo C.-C.J. (2020). Graph Representation Learning: A Survey. APSIPA Trans. Signal Inf. Process..

[57] Pedregosa F., Varoquaux G., Gramfort A., Michel V., Thirion B., Grisel O., Blondel M., Prettenhofer P., Weiss R., Dubourg V. (2011). Scikit-Learn: Machine Learning in Python. J. Mach. Learn. Res..

[58] Matys V., Kel-Margoulis O.V., Fricke E., Liebich I., Land S., Barre-Dirrie A., Reuter I., Chekmenev D., Krull M., Hornischer K. (2006). TRANSFAC and Its Module TRANSCompel: Transcriptional Gene Regulation in Eukaryotes. Nucleic Acids Res..

[59] Harrison P.W., Amode M.R., Austine-Orimoloye O., Azov A.G., Barba M., Barnes I., Becker A., Bennett R., Berry A., Bhai J. (2024). Ensembl 2024. Nucleic Acids Res..

[60] Csárdi G., Nepusz T. (2006). The Igraph Software Package for Complex Network Research. InterJournal—Complex Syst..

[61] Yu G., Wang L.-G., Han Y., He Q.-Y. (2012). clusterProfiler: An R Package for Comparing Biological Themes Among Gene Clusters. OMICS.

[62] Kanehisa M., Sato Y., Kawashima M., Furumichi M., Tanabe M. (2016). KEGG as a Reference Resource for Gene and Protein Annotation. Nucleic Acids Res..

[63] Gargano M.A., Matentzoglu N., Coleman B., Addo-Lartey E.B., Anagnostopoulos A.V., Anderton J., Avillach P., Bagley A.M., Bakštein E., Balhoff J.P. (2024). The Human Phenotype Ontology in 2024: Phenotypes around the World. Nucleic Acids Res..

[64] Kolberg L., Raudvere U., Kuzmin I., Adler P., Vilo J., Peterson H. (2023). G:Profiler—Interoperable Web Service for Functional Enrichment Analysis and Gene Identifier Mapping (2023 Update). Nucleic Acids Res..

